# Existential risk narratives about AI do not distract from its immediate harms

**DOI:** 10.1073/pnas.2419055122

**Published:** 2025-04-17

**Authors:** Emma Hoes, Fabrizio Gilardi

**Affiliations:** ^a^Department of Political Science, University of Zurich, Zurich 8050, Switzerland

**Keywords:** AI, survey experiments, AI safety

## Abstract

There is broad consensus that AI presents risks, but considerable disagreement about the nature of those risks. These differing viewpoints can be understood as distinct narratives, each offering a specific interpretation of AI’s potential dangers. One narrative focuses on doomsday predictions of AI posing long-term existential risks for humanity. Another narrative prioritizes immediate concerns that AI brings to society today, such as the reproduction of biases embedded into AI systems. A significant point of contention is that the “existential risk” narrative, which is largely speculative, may distract from the less dramatic but real and present dangers of AI. We address this “distraction hypothesis” by examining whether a focus on existential threats diverts attention from the immediate risks AI poses today. In three preregistered, online survey experiments (N = 10,800), participants were exposed to news headlines that either depicted AI as a catastrophic risk, highlighted its immediate societal impacts, or emphasized its potential benefits. Results show that i) respondents are much more concerned with the immediate, rather than existential, risks of AI, and ii) existential risk narratives increase concerns for catastrophic risks without diminishing the significant worries respondents express for immediate harms. These findings provide important empirical evidence to inform ongoing scientific and political debates on the societal implications of AI.

There is widespread agreement that AI poses significant risks, but the specific nature and scope of those risks is strongly disputed. Two perspectives stand out, each constituting a coherent narrative that attributes responsibility to particular aspects of AI’s development and application. First, the “existential risk” narrative posits that as AI systems advance, they could elude human control, leading to unforeseen and potentially catastrophic consequences for humanity (1). A frequently cited fact is that in a survey of machine learning researchers, the median respondent estimated a 5 percent probability that AI achieving human-level intelligence could result in outcomes such as human extinction (2); this estimate is now referred to as “p(doom).” The existential risk perspective was expressed prominently in the “Statement on AI risks” signed by leading AI scientists and CEOs in May 2023: “Mitigating the risk of extinction from AI should be a global priority alongside other societal-scale risks such as pandemics and nuclear war” (3). Second, the “immediate risks” narrative focuses on the tangible, current societal dangers posed by AI, building on the well-documented risks of earlier AI systems (4). It addresses issues such as the reproduction of societal biases in decision-making and harmful uses such as deepfake pornography or misinformation, arguing that these immediate problems are more urgent and relevant than speculative existential threats (5, 6). These two types of risks are not necessarily mutually exclusive (7). However, within the discourse, these narratives often appear as competing viewpoints (6, 8). A common critique from the immediate risks perspective is that the existential risk narrative, by focusing on future, speculative capabilities of AI systems, diverts attention from the current, demonstrated harms and aligns with the interests of technology companies (5). The debate has gained prominence following the release of ChatGPT at the end of 2022 (6), but it is rooted in enduring concerns that misguided fears about the impacts of AI may divert resources in unproductive directions (9). The media play a key role in this process, with alarmist media coverage of AI often aligning with existential risk themes (8).

Despite the importance of narratives about AI, there is insufficient knowledge regarding their impact (10). We provide empirical evidence on this question from three online survey experiments conducted in the United States (Studies 1, 2, and 3) and the United Kingdom (Study 2) (total N = 10,800). Participants were exposed to news headlines that either i) portrayed AI as a catastrophic risk, ii) highlighted its immediate societal impacts, or iii) underlined AI’s benefits in order to assess the impact of these narratives on the evaluation of different kinds of potential outcomes of AI’s development and application. Our findings show that while existential risk narratives increase assessments of potential catastrophic damages, they do *not* distract from concerns regarding AI’s immediate harms. In fact, our studies reveal that AI’s immediate harms consistently dominate public concern, with ethical issues, biases, misinformation, and job losses seen as the most pressing risks—persisting as top priorities even when participants are confronted with existential threats posed by AI. These results suggest that concerns about the negative implications of alarmist coverage of AI’s existential threats may be exaggerated, as our findings indicate no direct impact on public concern for the immediate risks of AI.

## Results

We employed a between-subjects experimental design with three main treatment groups. The treatments exposed participants to five headlines and corresponding lead texts emphasizing AI’s either existential or immediate risks, or AI’s positive capabilities. The headlines were generated by prompting ChatGPT to rewrite actual headlines and lead texts in the style of the New York Times to ensure ideological and stylistic homogeneity. This consistency in writing style aimed to ensure that any differences in participants’ responses are attributable to the content of the headlines. Participants in the “existential risks” group were shown only headlines related to existential threats, while those in the “immediate risks” group saw headlines focused solely on immediate dangers. The “positive outcomes” group was exposed exclusively to headlines highlighting AI’s beneficial capabilities. In contrast, the control group was not shown any headlines. A factor analysis demonstrates that existential and immediate risks load onto different factors, indicating they are distinct constructs. The headlines we used are reported in *SI Appendix*.

We preregistered 69 hypotheses: 18 in Study 1, 27 in Study 2, and 24 in Study 3. All changes between studies are fully documented in the relevant preregistrations for each study, with additional rationale provided in a Populated PAP (11) available in *SI Appendix*.^*^ Here, we evaluate the “distraction hypothesis” (preregistered as H1a in Study 1 and H2 in Studies 2 and 3) by focusing on the three key outcomes from Studies 1 and 2 combined in Study 3: i) *capability* assessing AI’s potential to generate specific effects; ii) *likelihood* measuring the probability of AI-related developments; and iii) *impact* evaluating the significance of these developments if they occur.

**Fig. 1.**
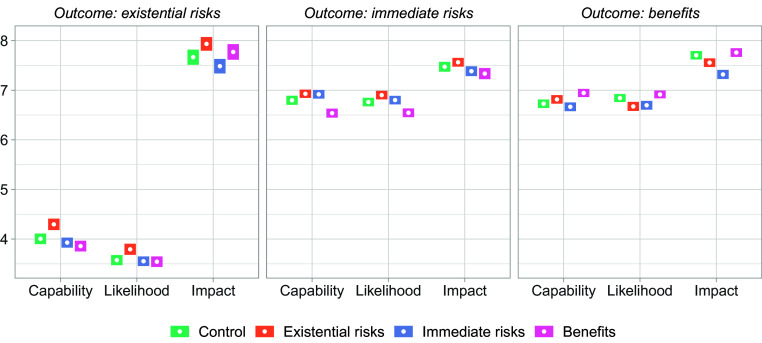
Rating of existential risks, immediate risks, and benefits of AI (1 to 10 scale) on three dimensions (capability, likelihood, impact) and across the control and three treatment groups, with 95% CIs (Study 3, N = 4,800). Concerns about AI’s capability and likelihood to cause immediate risks are higher than those regarding existential risks; both risks are viewed as roughly equal in terms of their potential impact.

Fig. 1 illustrates how respondents evaluated existential risks, immediate risks, and benefits of AI in terms of AI’s capability to produce these outcomes, as well as the likelihood and impact of each. Given that not all outcomes were included in Studies 1 and 2 (*Materials and Methods*), the figure focuses on respondents in Study 3 to ensure a clear visualization. Existential risks are rated significantly lower than immediate risks as well as benefits, both in terms of AI’s capability to produce these outcomes and the likelihood of their occurrence. Additionally, the perceived potential impact of existential risks is rated similarly to that of immediate risks and benefits. This suggests that respondents are not primarily concerned with the existential risks of AI and maintain a balanced view of immediate risks and benefits.

Linear regressions show that existential risk narratives increased the perceived *capability* of AI to cause existential risks. Significant effects were observed in Study 1 (Likert 1 to 5: *β* = 0.28, *P* = 0.00, *P* *adj*. = 0.00) and Study 3 (1 to 5: *β* = 0.25, *P* = 0.00, *P* *adj*. = 0.01), with weaker support for the 1 to 10 scale in Study 3 (*β* = 0.31, *P* = 0.04, *P* *adj*. = 0.25). By contrast, for the perceived capability of AI to cause immediate risks, significant positive effects of existential risk narratives were found in Study 1 (1 to 5: *β* = 0.18, *P* = 0.00, *P* *adj*. = 0.00), but the evidence weakened in Study 3, with diminished significance on the 1 to 5 scale (*β* = 0.12, *P* = 0.02, *P* *adj*. = 0.22) and no effect on the 1 to 10 scale (*β* = 0.14, *P* = 0.27, *P* *adj*. = 0.56). Regarding the effect of existential risk narratives on the perceived *impact* of immediate risks, results were mixed. Study 2 showed a trend toward decreased impact (1 to 10: *β* = −0.42, *P* = 0.00, *P* *adj*. = 0.051), but no significant effect was found in Study 3 (1 to 10: *β* = 0.11, *P* = 0.45, *P* *adj*. = 0.67). Findings for perceived *likelihood* of immediate risks were similarly inconsistent, with Study 2 suggesting an increase (1 to 10: *β* = 0.36, *P* = 0.03, *P* *adj*. = 0.37) and Study 3 showing no effect (1 to 10: *β* = 0.07, *P* = 0.59, P adj. = 0.71).

Overall, existential risk narratives consistently influence perceptions of AI’s capability to cause existential risks but show mixed effects on immediate harms. While some evidence suggests decreased perceived impact of immediate harms, other results indicate no significant effects or a tendency to increase their perceived likelihood. We thus do not find consistent evidence that existential risk narratives systematically distract from prioritizing immediate harms.

## Discussion

We provide evidence that existential risk narratives do not overshadow the immediate societal threats posed by AI. There are concerns that focusing on potential, long-term catastrophic risks diverts attention from more pressing issues, but our findings suggest otherwise. Exposure to narratives about existential risks increases awareness of these speculative threats, but it does not reduce concern for immediate harms. Worries for immediate risks remain consistently higher than for catastrophic risks regarding AI’s ability and likelihood to cause them and are roughly equal when considering their potential impact. Our findings contribute to ongoing debates on the societal implications of AI by highlighting the need for a balanced understanding of the risks it raises.

## Materials and Methods

Across studies, we measured a total of 19 outcomes: capability of causing existential risks 1), immediate risks 2), positive outcomes 3), conspiracies 4), importance of other societal issues 5), beliefs in falsehoods unrelated to AI 6), supporting a petition for responsible AI development 7), signing a petition 8), and ranking for impact 9). In Study 2, we introduced Likert scales for both likelihood and impact (10 to 15, measured for three risks), expanded the ranking to include likelihood 16), and added support for AI governance and policies 17), perceived power of AI 18), and feelings toward AI 19). In Study 3, no new outcomes were introduced, focusing exclusively on capability, likelihood, and impact. Scales for capability were adjusted to include both 1 to 5 and 1 to 10 formats to ensure comparability with Study 1. 1 to 10 scales for likelihood and impact introduced in Study 2 were retained for consistency. The three studies share the same design and were administered through Prolific Academic with samples balanced for ideology and gender. In Study 1 (fielded February 1, 2024) we recruited 3,000 US participants. In Study 2 (fielded May 21, 2024), we recruited 3,000 participants (1,500 UK, 1,500 US). In Study 3 (fielded June 13, 2024), we recruited 4,800 US participants. Respondents participated in only one study and were excluded from all subsequent studies. Capability was measured asking “Please rate each of the following statements based on how likely you think it is AI will cause or be capable of causing the described issue;” likelihood was measured asking “How likely do you believe it is that the following outcomes related to AI will occur within the next ten years?;” impact was measured asking “How significant would be the impact of each outcome, if it were to occur?.” For Study 2, we adapted the outcome measures to unpack the “capability” item from Study 1, as we realized that this measure may be conflating both likelihood and impact, thereby limiting its internal validity. The rationale for conducting Study 3 was twofold. First, we aimed to replicate the findings of both Study 1 and Study 2 to ensure the robustness and reliability of our results. Second, the results of Study 1 and Study 2 raised further questions about the extent to which participants assessed the three measures differently. This suggested a need to disentangle the dimensions of “capability,” “likelihood,” and “impact” more clearly. Respondents evaluated the capability, likelihood, and impact of AI’s existential risks, immediate risks, and benefits by rating one statement (Studies 1 and 2, randomized) or all statements (Study 3, randomized order) corresponding to each category (existential, immediate, benefits). All statements and more details on the study designs can be found in the corresponding preanalysis plans (Study 1: https://bit.ly/PAP-S1; Study 2: https://bit.ly/PAP-S2; Study 3: https://bit.ly/PAP-S3). Our study was approved by the University of Zurich’s PhF Ethics Committee (approval nr. 23.10.14). Informed consent was obtained from all participants.

## Supplementary Material

Appendix 01 (PDF)

## Data Availability

Data and code to reproduce all analyses have been deposited in Harvard Dataverse (DOI: 10.7910/DVN/5FASLG) (12).

## Footnotes

^*^The Populated PAP contains links to each preregistration as well as the results for all preregistered analyses.
