# Supplementary material for: Existential risk narratives about AI do not distract from its immediate harms
Source: Proc Natl Acad Sci U S A. 2025 Apr 17;122(16):e2419055122. doi: 10.1073/pnas.2419055122 (PMC12037001; doi:10.1073/pnas.2419055122)
Supplement: Supplementary file 1 — Appendix 01 (PDF) [file pnas.2419055122.sapp.pdf]

Existential Risk Narratives About Artificial Intelligence Do Not Distract From Its Immediate Harms: SI Appendix

Emma Hoes   Fabrizio Gilardi

March 13, 2025

Headlines Organized by Treatment

Treatment: Existential Risks

- **A.I. as a Global Threat: Industry Leaders Urge Caution and Regulation** Top executives from major A.I. companies, including OpenAI, Google DeepMind, and Anthropic, warn of the existential risks posed by artificial intelligence, likening its potential dangers to pandemics and nuclear wars. Over 350 experts call for urgent regulatory measures and global cooperation to mitigate A.I. threats.
- **CEOs Alarmed: AI's Existential Threat Within a Decade** A striking 42% of CEOs surveyed at the Yale CEO Summit believe artificial intelligence could pose a catastrophic threat to humanity in the next 5-10 years. This highlights the urgent need for strategic approaches to AI's rapid advancements.
- **AI's Dystopian Potential: Five Alarming Predictions from Top Researchers** Experts warn of AI's capacity to cause catastrophic harm, ranging from surpassing human intelligence to present-day biases in algorithms causing societal injustices.
- **AI's Existential Threat: A Call to Action from Leading Scientists** Sam Altman, CEO of OpenAI, and other top industry figures issue a stark warning about AI's potential to cause human extinction, emphasizing the need for urgent global prioritization.
- **AI's Potential Peril: Godfather of AI Has Grave Concerns Over Future of Humanity** Geoffrey Hinton, known as the 'godfather of AI', expresses concern about AI surpassing human intelligence, potentially leading to the 'end of people'.

Treatment: Imminent/Actual Risks

- **AI Revolution: A Tidal Wave of Job Disruption Looms on the Horizon** AI is poised to drastically reshape the global job market, prompting the need for proactive strategies from governments and corporations.

- **Generative AI Fuels Disinformation Surge, Amplifying Global Digital Oppression** A new report from Freedom House highlights the alarming use of generative AI by governments to spread disinformation and intensify censorship, posing a growing threat to internet freedom.
- **AI's Discrimination Dilemma: Severe Risks in Banking Sector Highlighted** The increasing use of AI in banking is raising concerns about human biases being amplified, potentially leading to discriminatory outcomes, urging the need for ethical AI practices.
- **AI Revolution Could Deepen Global Economic Inequality, Research Warns** A study reveals that AI could exacerbate the economic divide between developed and developing nations, prompting policymakers to focus on enhancing productivity and skills.
- **AI and Deepfakes: A Growing Threat in Cybersecurity Landscape** The rise of AI and deepfake technology is raising concerns about its misuse in fraudulent activities, calling for heightened awareness and training in cybersecurity.

Treatment: Positive Possibilities

- **DeepMind's AI Unveils Millions of New Material Possibilities, Revolutionizing Research** DeepMind's breakthrough AI can predict the properties of over 2.2 million new materials, revolutionizing fields such as electronics and solar cells.
- **AI Offers Breakthrough in Treating Rare Metabolic Disease in Children** Researchers have utilized AI to discover a promising new drug for cystinosis, a rare metabolic disorder, bringing new hope for effective treatments.
- **Innovative AI Model Revolutionizes Drug Synthesis, Boosting Efficiency and Sustainability** An AI model developed by leading researchers predicts optimal chemical alterations, significantly enhancing efficiency and sustainability in pharmaceutical development.
- **AI's Role in Tackling Climate Change: Harnessing Data for Effective Solutions** AI aids in analyzing vast climate data sets, helping to make informed predictions and deploy timely mitigation strategies for combating climate change.
- **Study Reveals AI's Positive Impact in Workplace Productivity and Employee Satisfaction** Research highlights the beneficial impact of AI in the workplace, showcasing increased productivity, enhanced customer experiences, and reduced employee turnover.

Survey Items Study 1, 2 and 3

Survey Study 1:

Consent form

General information

We appreciate your interest in participating in this study. You have been invited to take part as you have registered for this task on Prolific. Please note that you may only participate in this study if you are 18 years of age or over. Please read through the information provided and sign the consent form below, if you wish to proceed.

The aim of this study is to investigate your attitudes towards new digital technological developments, such as Artificial Intelligence (AI). As a participant, you will be asked to read one or several claims about such topics. This should take no more than 7 minutes. No background knowledge is required.

Do I have to take part?

Please note that your participation is voluntary. You may withdraw at any point during the questionnaire for any reason, before submitting your answers, by pressing the 'Exit' button / closing the browser. However, we only remunerate participants who complete the entire study.

Will I be compensated?

Yes. Participants who successfully complete the study will be paid at a flat rate of USD1.30 for their time.

How will your data be used?

Your answers will be completely anonymous, and we will take reasonable steps to keep them confidential.

Your data will be stored in a password-protected file and may be used in academic publications. Your IP address will not be stored. Research data will be stored for a minimum of three years after publication or public release.

Who will have access to your data?

The [blinded for peer review] is the data controller. Your information may be shared with other researchers internal to the project for data analysis and interpretation purposes. Responsible members of [blinded for peer review] and funders may also be given access to data for monitoring and/or auditing of the study to ensure we are complying with guidelines, or as otherwise required by law.

We would like your permission to use your anonymised data in future studies, and to share data with other researchers (e.g. in online databases). Any personal information that could

identify you will be removed or changed before files are shared with other researchers or results are made public.

Who is leading this study?

This study is part of the [blinded for peer review]. The principal researchers are [blinded for peer review].

What if there is a problem?

If you have a concern about any aspect of this project, please reach out to one of the lead researchers [blinded for peer review] who will do their best to answer your query. The researcher should acknowledge your concern within 10 working days and give you an indication of how they intend to deal with it. If you remain unhappy or wish to make a formal complaint, please contact the relevant Chair of the Research Ethics Committee at the [blinded for peer review]:

[contact info Ethics Committee]

The Chair will seek to resolve the matter in a reasonably expeditious manner.

[NEW PAGE]

Pre-treatment Items

Socio-demographics.

For statistical purposes, we need to know a bit about you

GENDER.
How do you describe yourself?

1. Male
2. Female
3. Other

AGE.
Which of the following categories includes your current age?

1. 17 or younger
2. 18 to 24
3. 25 to 34
4. 35 to 44
5. 45 to 54
6. 55 to 64
7. 65+

RACE.
How do you describe yourself? (Please check the one option that best describes you)
o American Indian or Alaskan Native (1)
o Hawaiian or other Pacific islander (2)
o Asian or Asian American (3)
o Black or African American (4)
o Hispanic or Latino (5)
o Non-Hispanic White (6)
o Other (7)

EDUCAT.
What is the highest level of school you have completed?
a) None, or grades 1-8
b) High school incomplete (grades 9-11)
c) High school graduate (grade 12 or GED certificate)
d) Technical, trade or vocational school AFTER high school
e) Some college, no 4-year degree (includes associate degree)
f) College graduate (B.S., B.A., or other 4-year degree)
g) Post-graduate training/professional school after college (toward a Master's degree or Ph.D., Law or Medical school)

IDEO.
How would you rate yourself on this scale?

1. Very liberal
2. Somewhat liberal
3. Middle of the road
4. Somewhat conservative
5. Very conservative

POLINT.
How interested are you in politics?
1. Not at all interested
2. Not very interested
3. Somewhat interested
4. Very interested
5. Extremely interested

ATTN.
To check whether you are reading the questions, click on the second answer from the top.
- Never (1)
- Last year (2)
- Last month (3)
- Last week (4)
- Yesterday (5)
- Other (6) ____________________

AI Exposure and Use.

Artificial Intelligence (AI) refers to the creation of computer systems or software that can perform tasks typically requiring human intelligence. These tasks include understanding language, recognizing images, solving problems, and making decisions. AI can range from simple programs, like those recommending movies based on your preferences, to more complex ones, such as self-driving cars or virtual assistants like Siri or Alexa. AI technology is designed to learn and adapt based on new information or experiences, mimicking the way humans think and learn. Additionally, generative AI, a subset of AI, focuses on creating new content, such as text, images, or music, based on training data, showcasing creativity and innovation in machine learning. Tools such as ChatGPT are an example of generative AI.

In this study, when we talk or ask about AI, we are referring to this definition.

Have you heard about AI before?

1. Yes
2. No
3. Don't know

Where have you heard about AI? Please tick all that apply

Category 1:
In the news on TV
In the news online
In the news on the radio
In the news on print

Category 2:
On YouTube
On Twitter
On Instagram
On Facebook
On TikTok
On Snapchat
On Reddit
On WhatsApp
On Messenger
On Telegram

Category 3:
During offline discussions with friends, family, colleagues, etc.

In relation to what topic have you heard about AI? Tick all that apply
Randomize order of options, but 'other' should always be last (thus not included in the randomization)

education
economy
entertainment
healthcare
coding/data science/computer science
journalism
malicious content (bots, trolls, fake news, hate speech, etc.)
art (novels, poems, jokes, etc.)
personal correspondence (emails, etc.)

if 'other': open ended question

In general, when you heard about AI, was it in positive terms or negative terms?

1. Negative
2. Neutral
3. Positive

Have you used AI software before?

1. Yes
2. No

For what purpose? [If yes; open text box]

INTRO TREATMENT.

On the next pages you will be presented with several recent news headlines about Artificial Intelligence. We would like you to read each headline carefully. At the end of the survey we will ask you some questions about them.

Treatment: Existential Risks

A.I. as a Global Threat: Industry Leaders Urge Caution and Regulation Top executives from major A.I. companies, including OpenAI, Google DeepMind, and Anthropic, warn of the existential risks posed by artificial intelligence, likening its potential dangers to pandemics and nuclear wars. In a rare consensus, over 350 experts call for urgent regulatory measures and global cooperation to mitigate A.I. threats, underscoring the technology's rapid advancement and its unforeseen societal impacts.

CEOs Alarmed: AI's Existential Threat Within a Decade A striking 42% of CEOs surveyed at the Yale CEO Summit believe artificial intelligence could pose a catastrophic threat to humanity in the next 5-10 years. The survey, reflecting views from diverse industries, reveals a split among business leaders on AI's potential dangers versus its opportunities, highlighting

the urgent need for a deeper understanding and strategic approach to AI's rapid advancements.

AI's Dystopian Potential: Five Alarming Predictions from Top Researchers Experts warn of artificial intelligence's capacity to cause catastrophic harm, drawing parallels to species extinction and global disruption. These scenarios range from AI surpassing human intelligence and manipulating resources to present-day biases in algorithms causing societal injustices, illustrating both immediate and future threats posed by unregulated AI development.

AI's Existential Threat: A call to action from Leading Scientists
Sam Altman, CEO of OpenAI, and other top industry figures issue a stark warning about AI's potential to cause human extinction. This unified statement, emphasizing the urgent need for global prioritization akin to addressing pandemics and nuclear war, reflects a growing consensus among scientists and tech leaders about the imminent risks of unregulated AI development.

AI's Potential Peril: Godfather of AI has Grave Concerns Over Future of Humanity
Geoffrey Hinton, renowned as the 'godfather of AI', expresses his alarm about artificial intelligence surpassing human intelligence, potentially leading to 'the end of people.' While his warnings contrast with the views of other experts who emphasize AI's immediate challenges and benefits, the debate highlights the crucial need for responsible development and regulation of AI technologies.

Treatment: Imminent/Actual Risks

AI Revolution: A Tidal Wave of Job Disruption Looms on the Horizon
The impending wave of artificial intelligence is poised to drastically reshape the global job market, with predictions of significant job displacement and transformation. Amidst the potential for both economic upheaval and unprecedented productivity gains, the need for proactive government and corporate strategies to manage this transition becomes increasingly vital.

Generative AI Fuels Disinformation Surge, Amplifying Global Digital Oppression A new report from Freedom House highlights the alarming use of generative AI by governments worldwide to spread disinformation and intensify internet censorship. This evolving digital strategy, encompassing AI-generated texts, images, and videos, poses a growing threat to internet freedom and public discourse, marking a troubling trend in the manipulation of online information.

AI's Discrimination Dilemma: Severe Risks in Banking Sector Highlighted The increasing use of AI in banking and financial services is raising serious concerns about the amplification of existing human biases, potentially leading to discriminatory outcomes. Experts emphasize the need for comprehensive datasets and unbiased development teams to counter this trend, underscoring the urgency for effective regulatory measures and ethical AI practices in the financial industry.

AI Revolution Could Deepen Global Economic Inequality, Research Warns A study reveals that the rise of artificial intelligence and robotics could exacerbate the economic divide between developed and developing nations, threatening to channel more investment towards advanced economies and potentially replace the labor force in less developed countries. This shift urges policymakers in developing nations to focus on enhancing productivity and skills to avoid being left behind in the global AI-driven transformation.

AI and Deepfakes: A Growing Threat in Cybersecurity Landscape The proliferation of AI and deepfake technology is raising alarms in the IT sector, with concerns about its misuse in fraudulent activities and its impact on businesses. While AI holds the potential for positive contributions, its increasing sophistication in creating synthetic media poses significant risks, calling for heightened awareness and training in cybersecurity measures to combat these emerging challenges.

Treatment: Positive Possibilities

DeepMind's AI Unveils Millions of New Material Possibilities, Revolutionizing Research DeepMind's latest breakthrough in AI, with the ability to predict the properties of over 2.2 million new materials, marks a significant leap forward in materials science. This innovation could pave the way for advancements in diverse fields such as electronics, batteries, and solar cells, potentially transforming the landscape of modern technology and manufacturing.

AI Offers Breakthrough in Treating Rare Metabolic Disease in Children Leveraging the power of big data, advanced algorithms, and high-performance computing, researchers have utilized artificial intelligence to discover a promising new drug for cystinosis, a rare metabolic disorder. This groundbreaking research, funded by the Swiss National Science Foundation and led by the University of Zurich, brings new hope for effective treatment options for affected children worldwide.

Innovative AI Model Revolutionizes Drug Synthesis, Boosting Efficiency and Sustainability Researchers from the Ludwig Maximilian University of Munich, ETH Zurich, and Roche Pharma have developed a groundbreaking AI model capable of predicting the optimal chemical alterations in drug molecules. This advancement promises to reduce laboratory experiments significantly, enhancing the efficiency and sustainability of chemical synthesis in pharmaceutical development. The model accelerates the creation of more effective drug variants, marking a significant leap in medicinal chemistry.

AI's Role in Tackling Climate Change: Harnessing Data for Effective Solutions Experts at the Johns Hopkins Institute for Assured Autonomy highlight the significant potential of AI in addressing the complexities of climate change. By analyzing vast climate data sets, AI aids in making informed predictions and deploying timely mitigation strategies. The technology's application ranges from understanding oceanic changes to monitoring environmental shifts via satellites, offering a transformative approach to combating one of the most pressing global challenges.

Study Reveals AI's Positive Impact in Workplace Productivity and Employee Satisfaction

Recent research by a team of economists, including Stanford's Erik Brynjolfsson, highlights the beneficial impact of AI in the workplace, challenging the notion of AI as a job eliminator. Their study, focusing on AI in customer service, demonstrates increased worker productivity, enhanced customer experiences, and reduced employee turnover, showcasing AI as a valuable tool in augmenting human capabilities and improving overall workplace dynamics.

Post-treatment Items

MANIP CHECK.
You were just presented with some news headlines. Please tell us to what extent you agree of disagree with the following statement:

- The headlines I just saw were mostly about existential risks AI may bring
- The headlines I just saw were mostly about the positive potential that AI holds
- The headlines I just saw were mostly about the imminent risks AI poses today

Participants saw the statement corresponding to the treatment group to which they were assigned.

ATTITUDES.
Next, you will see a list of various risks and possibilities associated with Artificial Intelligence (AI). For each item, please rate how likely you think it is that AI will cause or be capable of causing this issue. Your insights are valuable in understanding public perception of AI's potential impact.

Please rate each of the following statements based on how likely you think AI will cause or be capable of causing the described issue:

Each respondents saw one statement (randomized) from each category; they thus saw 4 in total

1. Existential Risks
   - AI leading to a global catastrophic event.
   - AI making humans obsolete.
   - AI autonomously starting a war.
   - AI causing a significant environmental disaster.

2. Immediate/Actual Risks
   - AI leading to significant job losses in certain sectors.
   - AI being used in mass surveillance systems.
   - AI increasing the spread of misinformation online.
   - AI exacerbating biases in decision-making processes.

3. Positive Possibilities
   - AI improving healthcare through better diagnosis and treatment.
   - AI enhancing education through personalized learning.

- AI reducing human error in critical tasks.
- AI contributing to solving complex environmental issues.

4. Conspiracy theories
   - AI surpassing human intelligence and taking over the world, either displacing or enslaving humanity.
   - AI being developed in secret by governments or corporations as a form of superintelligent AI for unknown purposes.
   - AI being a derivative of alien technology, not originally invented by humans.

Rating Scale:
1. Very Unlikely
2. Unlikely
3. Neutral
4. Likely
5. Very Likely

SEVERITY.

Please rank the likely impact (severity) of the following four threats associated to AI over the next 10 years, in the order from (1) most to least (3) severe

- Global catastrophic events
- Current socioeconomic and ethical problems
- Undisclosed AI developments

SPILLOVERS.

Importance of other societal issues.

In your personal opinion, to what extent do you agree or disagree that the issues below demand a lot of political attention at present?

- Economy in general
- High cost of living/Inflation
- Unemployment/Jobs
- Immigration
- Poverty/Hunger/Homelessness
- Crime/Violence
- Race relations/Racism
- Healthcare

Rating Scale:
1. Strongly agree
2. Agree
3. Somewhat agree

4.. Neither agree nor disagree
5. Somewhat disagree
6. Disagree
7. Strongly disagree

Belief in falsehoods unrelated to AI.

Participants had to rate three false news headlines which were randomly drawn from this pool of recent headlines:
https://osf.io/2jrwa/?view_only=41d1004e9f7f45f1ab3a14150f00999f

BEHAVIORS.

Please read the information below carefully.

Approximately six months ago there was a movement initiated by certain individuals advocating for a pause in the development of highly advanced AI technologies. This pause, proposed for a duration of six months, aims at halting the creation of AI systems more sophisticated than the current top-tier models. The purpose of this petition is to ensure the safety of these AI systems, to prevent potential harm, and to establish regulations governing their usage. The ultimate objective of this petition is to enhance the safety and efficacy of AI technologies, while also preparing for the significant impacts they may have on employment and societal structures.

First, we are interested in knowing if you would support this petition.

Petition_Support

Would you support this petition?

[Yes]
[No]

Petition_Sign

Next, we would like to know whether you intend to sign the petition. Your participation is entirely voluntary. Participating or withdrawing will have no consequences for your remuneration. Clicking on the link below will open a new window in your browser, indicates to us that you intend to sign the petition, and forwards you to the debriefing of this study. If you do not intend to sign the petition, please ignore the link and press the arrow at the bottom of this page to continue to the debriefing.

Link to the petition: https://shorturl.at/gmAHZ

DEBRIEFING.

Thank you for your participation in this research study. Now that you completed or have ended your participation, we will provide you with some additional information about the purposes of this study.

What you should know about this study

The main purpose of this study was for us to observe how participants feel about new technological developments, such as AI and ChatGPT. Participants in this study have been exposed to various headlines about AI, some highlighting the more positive aspects of AI development, some highlighting more negative aspects of it. It is important to realize that the headlines you have been exposed to, may not be an accurate representation of the variety in which the media covers AI related issues.

If you have questions

The main researchers conducting this study are [blinded for peer review]. If you have questions, you may contact one of the main researchers. If you have any questions or concerns regarding your rights as a research participant in this study, you may contact the Ethics Committee.

[contact info Ethics Committee]

Survey Study 2

Consent form (identical to Study 1)

<u>Pre-treatment Items</u>

Socio-demographics.
For statistical purposes, we need to know a bit about you

GENDER.
How do you describe yourself?
1. Male
2. Female
3. Other

AGE.
Which of the following categories includes your current age?
1. 17 or younger
2. 18 to 24
3. 25 to 34
4. 35 to 44
5. 45 to 54
6. 55 to 64
7. 65+

RACE. (US)

How do you describe yourself? (Please check the one option that best describes you) o American Indian or Alaskan Native (1)
o Hawaiian or other Pacific islander (2)
o Asian or Asian American (3)
o Black or African American (4)
o Hispanic or Latino (5)
o Non-Hispanic White (6)
o Other (7)

RACE. (UK)
o White (1)
o Mixed or Multiple ethnics backgrounds (2)
o Black, African, Carribean, or Black British (3)
o Asian or Asian British (4)
o Other (5)

EDUCAT.
What is the highest level of school you have completed?
a) None, or grades 1-8
b) High school incomplete (grades 9-11)

c) High school graduate (grade 12 or GED certificate)
d) Technical, trade or vocational school AFTER high school
e) Some college, no 4-year degree (includes associate degree)
f) College graduate (B.S., B.A., or other 4-year degree)
g) Post-graduate training/professional school after college (toward a Master's degree or Ph.D., Law or Medical school)

IDEO.
How would you rate yourself on this scale?
1. Very liberal
2. Somewhat liberal
3. Middle of the road
4. Somewhat conservative
5. Very conservative

POLINT.
How interested are you in politics?
1. Not at all interested
2. Not very interested
3. Somewhat interested
4. Very interested
5. Extremely interested

ATTN.
To check whether you are reading the questions, click on the second answer from the top.
- Never (1)
- Last year (2)
- Last month (3)
- Last week (4)
- Yesterday (5)
- Other (6) ______________________

AI Exposure and Use.
Artificial Intelligence (AI) refers to the creation of computer systems or software that can perform tasks typically requiring human intelligence. These tasks include understanding language, recognizing images, solving problems, and making decisions. AI can range from simple programs, like those recommending movies based on your preferences, to more complex ones, such as self-driving cars or virtual assistants like Siri or Alexa. AI technology is designed to learn and adapt based on new information or experiences, mimicking the way humans think and learn. Additionally, generative AI, a subset of AI, focuses on creating new content, such as text, images, or music, based on training data. Tools such as ChatGPT are an example of generative AI.
In this study, when we talk or ask about AI, we are referring to this definition.

Have you heard about AI before?
1. Yes
2. No

3. Don’t know

In general, when you heard about AI, was it in positive terms or negative terms?
1. Negative
2. Neutral
3. Positive

Have you used AI software, like ChatGPT, before?
1. Yes
2. No

AI Knowledge

Choose the option(s) that is correct regarding artificial intelligence (AI): Select all that apply.
- - AI involves methods that enable systems to learn on their own without direct programming. (correct)
- - Machine learning is a type of artificial intelligence. (correct)
- - AI is a software, machine, or computer that is expected to eventually replicate the human mind. (incorrect)
- - None of the above. (incorrect)
- - I don’t know.

Which statement about artificial intelligence is true?
A) AI is primarily used for solving complex mathematical computations.. (false) B) AI involves creating systems that can learn and make decisions. (true)
C) All AI systems are fully autonomous robots. (false)
D) AI was invented in the 21st century. (false)
E) None of the above. (false)

AI Risks Awareness
To what extent do you consider yourself to be aware of how AI may impact society?
1. Not at all aware
2. Not very aware
3. Somewhat aware
4. Very aware
5. Extremely aware

INTRO TREATMENT.
On the next pages you will be presented with several recent news headlines followed by a lead text about Artificial Intelligence. We would like you to read each headline carefully, and let us know whether you - hypothetically - would like to read the entire article if you would come across this headline in real life.

At the end of the survey we will ask you some more questions about them.

[Below each headline: Would you like to read the full article? Answer options: Yes / No]

Treatment: Existential Risks
A.I. as a Global Threat: Industry Leaders Urge Caution and Regulation Top executives from major A.I. companies, including OpenAI, Google DeepMind, and Anthropic, warn of the existential risks posed by artificial intelligence, likening its potential dangers to pandemics and nuclear wars. In a rare consensus, over 350 experts call for urgent regulatory measures and global cooperation to mitigate A.I. threats, underscoring the technology's rapid advancement and its unforeseen societal impacts.

CEOs Alarmed: AI's Existential Threat Within a Decade A striking 42% of CEOs surveyed at the Yale CEO Summit believe artificial intelligence could pose a catastrophic threat to humanity in the next 5-10 years. The survey, reflecting views from diverse industries, reveals a split among business leaders on AI's potential dangers versus its opportunities, highlighting the urgent need for a deeper understanding and strategic approach to AI's rapid advancements.

AI's Dystopian Potential: Five Alarming Predictions from Top Researchers Experts warn of artificial intelligence's capacity to cause catastrophic harm, drawing parallels to species extinction and global disruption. These scenarios range from AI surpassing human intelligence and manipulating resources to present-day biases in algorithms causing societal injustices, illustrating both immediate and future threats posed by unregulated AI development.

AI's Existential Threat: A call to action from Leading Scientists
Sam Altman, CEO of OpenAI, and other top industry figures issue a stark warning about AI's potential to cause human extinction. This unified statement, emphasizing the urgent need for global prioritization akin to addressing pandemics and nuclear war, reflects a growing consensus among scientists and tech leaders about the imminent risks of unregulated AI development.

AI's Potential Peril: Godfather of AI has Grave Concerns Over Future of Humanity Geoffrey Hinton, renowned as the 'godfather of AI', expresses his alarm about artificial intelligence surpassing human intelligence, potentially leading to 'the end of people.' While his warnings contrast with the views of other experts who emphasize AI's immediate challenges and benefits, the debate highlights the crucial need for responsible development and regulation of AI technologies.

Treatment: Imminent/Actual Risks
AI Revolution: A Tidal Wave of Job Disruption Looms on the Horizon The impending wave of artificial intelligence is poised to drastically reshape the global job market, with predictions of significant job displacement and transformation. Amidst the potential for both economic upheaval and unprecedented productivity gains, the need for proactive government and corporate strategies to manage this transition becomes increasingly vital.

Generative AI Fuels Disinformation Surge, Amplifying Global Digital Oppression A new report from Freedom House highlights the alarming use of generative AI by governments worldwide to spread disinformation and intensify internet censorship. This evolving digital

strategy, encompassing AI-generated texts, images, and videos, poses a growing threat to internet freedom and public discourse, marking a troubling trend in the manipulation of online information.

AI's Discrimination Dilemma: Severe Risks in Banking Sector Highlighted The increasing use of AI in banking and financial services is raising serious concerns about the amplification of existing human biases, potentially leading to discriminatory outcomes. Experts emphasize the need for comprehensive datasets and unbiased development teams to counter this trend, underscoring the urgency for effective regulatory measures and ethical AI practices in the financial industry.

AI Revolution Could Deepen Global Economic Inequality, Research Warns A study reveals that the rise of artificial intelligence and robotics could exacerbate the economic divide between developed and developing nations, threatening to channel more investment towards advanced economies and potentially replace the labor force in less developed countries. This shift urges policymakers in developing nations to focus on enhancing productivity and skills to avoid being left behind in the global AI-driven transformation.

AI and Deepfakes: A Growing Threat in Cybersecurity Landscape The proliferation of AI and deepfake technology is raising alarms in the IT sector, with concerns about its misuse in fraudulent activities and its impact on businesses. While AI holds the potential for positive contributions, its increasing sophistication in creating synthetic media poses significant risks, calling for heightened awareness and training in cybersecurity measures to combat these emerging challenges.

Treatment: Positive Possibilities

DeepMind's AI Unveils Millions of New Material Possibilities, Revolutionizing Research DeepMind's latest breakthrough in AI, with the ability to predict the properties of over 2.2 million new materials, marks a significant leap forward in materials science. This innovation could pave the way for advancements in diverse fields such as electronics, batteries, and solar cells, potentially transforming the landscape of modern technology and manufacturing.

AI Offers Breakthrough in Treating Rare Metabolic Disease in Children Leveraging the power of big data, advanced algorithms, and high-performance computing, researchers have utilized artificial intelligence to discover a promising new drug for cystinosis, a rare metabolic disorder. This groundbreaking research, funded by the Swiss National Science Foundation and led by the University of Zurich, brings new hope for effective treatment options for affected children worldwide.

Innovative AI Model Revolutionizes Drug Synthesis, Boosting Efficiency and Sustainability Researchers from the Ludwig Maximilian University of Munich, ETH Zurich, and Roche Pharma have developed a groundbreaking AI model capable of predicting the optimal chemical alterations in drug molecules. This advancement promises to reduce laboratory experiments significantly, enhancing the efficiency and sustainability of chemical synthesis in pharmaceutical development. The model accelerates the creation of more effective drug variants, marking a significant leap in medicinal chemistry.

AI's Role in Tackling Climate Change: Harnessing Data for Effective Solutions Experts at the Johns Hopkins Institute for Assured Autonomy highlight the significant potential of AI in addressing the complexities of climate change. By analyzing vast climate data sets, AI aids in making informed predictions and deploying timely mitigation strategies. The technology's application ranges from understanding oceanic changes to monitoring environmental shifts via satellites, offering a transformative approach to combating one of the most pressing global challenges.

Study Reveals AI's Positive Impact in Workplace Productivity and Employee Satisfaction Recent research by a team of economists, including Stanford's Erik Brynjolfsson, highlights the beneficial impact of AI in the workplace, challenging the notion of AI as a job eliminator. Their study, focusing on AI in customer service, demonstrates increased worker productivity, enhanced customer experiences, and reduced employee turnover, showcasing AI as a valuable tool in augmenting human capabilities and improving overall workplace dynamics.

Post-treatment Items

MANIP CHECK.

You were just presented with some news headlines. Please tell us to what extent you agree of disagree with the following statement:
- The headlines I just saw were mostly about existential risks AI may bring - The headlines I just saw were mostly about the positive potential that AI holds - The headlines I just saw were mostly about the imminent risks AI poses today
Participants saw the statement corresponding to the treatment group to which they were assigned.

ATTITUDES - LIKERT 1 - 10

Next, you will see a list of various outcomes associated with Artificial Intelligence (AI). For each item listed, we would like you to answer the following question:

1. How likely do you believe that the following outcomes related to Artificial Intelligence will occur within the next ten years?

Remember, there are no right or wrong answers. Your perspectives are invaluable for understanding how the public perceives the potential impacts of AI
Please assess each statement by estimating the probability of occurrence of each listed outcome.

OR

Next, you will see a list of various outcomes associated with Artificial Intelligence (AI). For each item listed, we would like you to answer the following question:

2. How significant would be the impact of each outcome, if it were to occur?

Remember, there are no right or wrong answers. Your perspectives are invaluable for understanding how the public perceives the potential impacts of AI.

Please assess each statement by estimating the potential impact of each listed outcome.

Each respondents sees one statement (randomized) from each category; they thus see 5 in total

1. Existential Risks
- AI leading to a global catastrophic event.
- AI making humans obsolete.
- AI autonomously starting a war.
- AI causing a significant environmental disaster.
2. Immediate/Actual Risks
- AI leading to significant job losses in certain sectors.
- AI being used in mass surveillance systems.
- AI increasing the spread of misinformation online.
- AI making decision by organizations (public and private) more biased .
3. Positive Possibilities
- AI improving healthcare through better diagnosis and treatment.
- AI enhancing education through personalized learning.
- AI reducing human error in critical tasks.
- AI contributing to solving complex environmental issues.
4. Conspiracy theories
- AI surpassing human intelligence and taking over the world
- AI being developed in secret by governments or corporations as a form of superintelligent AI for unknown purposes.
- AI being a derivative of alien technology, not originally invented by humans. - AI containing a hidden code that predicts future events, known only to a selet few who use it to manipulate global markets and politics
Rating Scale:
Likelihood
1. Extremely Unlikely
2. Very Unlikely
3. Moderately Unlikely
4. Slightly Unlikely
5. Neutral
6. Slightly Likely
7. Moderately Likely
8. Likely
9. Very Likely
10. Extremely Likely

Impact

1. Extremely Insignificant
2. Very Insignificant

3. Moderately Insignificant
4. Slightly Insignificant
5. Neutral
6. Slightly Significant
7. Moderately Significant
8. Significant
9. Very Significant
10. Extremely Significant

ATTITUDES - RANKING

You will now be presented with a list of 12 AI-related outcomes. Some of these may be familiar from earlier questions. Please rank each risk on the following scales:

1. Likelihood of Occurrence:

- "Rank the likelihood of each AI-related outcome occurring within the next ten years. Assign a rank of (1) for the outcome you believe is most likely to occur and a rank of (12) for the outcome you believe is least likely to occur."

OR

You will now be presented with a list of 12 AI-related outcomes. Some of these may be familiar from earlier questions. Please rank each risk on the following scales:

2. Significance of Outcome:

- "Rank the significance of the impact if each AI-related outcome were to occur. Use a rank of (1) to indicate the outcome with the most significant consequences and a rank of (12) for the outcome with the least significant consequences."

PERCEIVED POWER OF AI

On a scale of 1-7, how powerful do you think Artificial Intelligence systems are?

1. Not powerful at all
2. Slightly powerful
3. Somewhat powerful
4. Moderately powerful
5. Quite powerful
6. Very powerful
7. Extremely powerful

How do you feel about the power of Artificial Intelligence? Please select where your feelings fall on this scale:

1. Very fearful
2. Somewhat fearful
3. Slightly fearful
4. Neutral
5. Slightly hopeful
6. Somewhat hopeful
7. Very hopeful

AI Governance

Respondents see 1 of each category (governance, AI act, US bill of rights) randomly

1. To what extent do you support the implementation of strict regulations on the development and use of artificial intelligence to ensure public safety? Please rate your support on a scale from 1 (Strongly Disagree) to 7 (Strongly Agree).
2. How important do you think it is for international organizations to play a role in overseeing AI development globally? Please rate from 1 (Not at all important) to 7 (Extremely important)."
3. Do you agree that there should be transparent public reporting of AI testing and outcomes to ensure accountability? Rate your agreement on a scale from 1 (Strongly Disagree) to 7 (Strongly Agree)."
4. How strongly do you support the creation of ethical standards for AI that all developers and companies must follow? Please express your level of support on a scale from 1 (Strongly Disagree) to 7 (Strongly Agree).
5. Do you believe that consumers should have the right to know and decide whenever AI is used in decision-making processes that affect them? Please rate your agreement from 1 (Strongly Disagree) to 7 (Strongly Agree)."

AI Policies

Based on AI Act

1. Do you support banning the use of AI to recognize faces in real time in public places, with some exceptions like national security? Rate your support from 1 (Strongly Disagree) to 7 (Strongly Agree).
2. Do you support requiring safety and legal checks on AI systems before they are sold? Please rate your support from 1 (Strongly Disagree) to 7 (Strongly Agree).
3. Do you support setting up special areas where AI can be tested under close watch? Indicate your support on a scale from 1 (Strongly Disagree) to 7 (Strongly Agree).
4. Do you support making AI systems that are used in important areas, like schools or important services, easy to understand and transparent? Please rate from 1 (Strongly Disagree) to 7 (Strongly Agree).

Based on US' AI Bill of Rights

1. AI System Safety and Effectiveness
- "Do you think AI systems should be tested to make sure they are safe and work well before they are used? Please rate from 1 (Strongly Disagree) to 7 (Strongly Agree)."

2. Preventing Discrimination in AI
- "How important is it to you that AI systems do not discriminate against people? Rate from 1 (Not at all important) to 7 (Extremely important)."

3. Data Privacy in AI
- "Do you agree that people should have strong protection against misuse of their data and control over their personal information used by AI systems? Rate your agreement from 1 (Strongly Disagree) to 7 (Strongly Agree)."

4. Transparency of AI Use
- "Should people be clearly told when an AI system is used and understand how it affects decisions about them? Please rate your agreement from 1 (Strongly Disagree) to 7 (Strongly Agree)."

DEBRIEFING.

Thank you for your participation in this research study. Now that you completed or have ended your participation, we will provide you with some additional information about the purposes of this study.

What you should know about this study
The main purpose of this study was for us to observe how participants feel about new technological developments, such as AI and ChatGPT. Participants in this study have been exposed to various headlines about AI, some highlighting the more positive aspects of AI development, some highlighting more negative aspects of it. It is important to realize that the headlines you have been exposed to, may not be an accurate representation of the variety in which the media covers AI related issues.
If you have questions
The main researchers conducting this study are [names and contact details of researchers]. If you have questions, you may contact one of the main researchers. If you have any questions or concerns regarding your rights as a research participant in this study, you may contact the Ethics Committee.
[contact details Ethics Committee]

Survey Study 3

Consent form (identical to Study 1 and 2)

<u>**Pre-treatment Items**</u>

Socio-demographics.

For statistical purposes, we need to know a bit about you

GENDER.
How do you describe yourself?

1. Male
2. Female
3. Other

AGE.
Which of the following categories includes your current age?

1. 17 or younger
2. 18 to 24
3. 25 to 34
4. 35 to 44
5. 45 to 54
6. 55 to 64
7. 65+

RACE. (US)

How do you describe yourself? (Please check the one option that best describes you)
o American Indian or Alaskan Native (1)
o Hawaiian or other Pacific islander (2)
o Asian or Asian American (3)
o Black or African American (4)
o Hispanic or Latino (5)
o Non-Hispanic White (6)
o Other (7)

EDUCAT.
What is the highest level of school you have completed?
a) None, or grades 1-8
b) High school incomplete (grades 9-11)
c) High school graduate (grade 12 or GED certificate)
d) Technical, trade or vocational school AFTER high school
e) Some college, no 4-year degree (includes associate degree)
f) College graduate (B.S., B.A., or other 4-year degree)
g) Post-graduate training/professional school after college (toward a Master's degree or Ph.D., Law or Medical school)

IDEO.
How would you rate yourself on this scale?

1. Very liberal
2. Somewhat liberal
3. Middle of the road
4. Somewhat conservative
5. Very conservative

POLINT.
How interested are you in politics?
1. Not at all interested
2. Not very interested
3. Somewhat interested
4. Very interested
5. Extremely interested

ATTN.
To check whether you are reading the questions, click on the second answer from the top.
- Never (1)
- Last year (2)
- Last month (3)
- Last week (4)
- Yesterday (5)
- Other (6) ____________________

AI Exposure and Use.

Artificial Intelligence (AI) refers to the creation of computer systems or software that can perform tasks typically requiring human intelligence. These tasks include understanding language, recognizing images, solving problems, and making decisions. AI can range from simple programs, like those recommending movies based on your preferences, to more complex ones, such as self-driving cars or virtual assistants like Siri or Alexa. AI technology is designed to learn and adapt based on new information or experiences, mimicking the way humans think and learn. Additionally, generative AI, a subset of AI, focuses on creating new content, such as text, images, or music, based on training data. Tools such as ChatGPT are an example of generative AI.

In this study, when we talk or ask about AI, we are referring to this definition.

Have you heard about AI before?

1. Yes
2. No
3. Don't know

In general, when you heard about AI, was it in positive terms or negative terms?

1. Negative
2. Neutral
3. Positive

Have you used AI software, like ChatGPT, before?

1. Yes
2. No

AI Knowledge

Choose the option(s) that is correct regarding artificial intelligence (AI): Select all that apply.

- - AI involves methods that enable systems to learn on their own without direct programming. (correct)
- - Machine learning is a type of artificial intelligence. (correct)
- - AI is a software, machine, or computer that is expected to eventually replicate the human mind. (incorrect)
- - None of the above. (incorrect)
- - I don't know.

Which statement about artificial intelligence is true?

A) AI is primarily used for solving complex mathematical computations.. (false)
B) AI involves creating systems that can learn and make decisions. (true)
C) All AI systems are fully autonomous robots. (false)
D) AI was invented in the 21st century. (false)
E) None of the above. (false)

AI Risks Awareness

To what extent do you consider yourself to be aware of how AI may impact society?

1. Not at all aware
2. Not very aware
3. Somewhat aware
4. Very aware
5. Extremely aware

INTRO TREATMENT.

On the next pages you will be presented with several recent news headlines followed by a lead text about Artificial Intelligence. We would like you to read each headline carefully, and let us know whether you - hypothetically - would like to read the entire article if you would come across this headline in real life.

At the end of the survey we will ask you some more questions about them.

[Below each headline: Would you like to read the full article? Answer options: Yes / No]

Treatment: Existential Risks

A.I. as a Global Threat: Industry Leaders Urge Caution and Regulation Top executives from major A.I. companies, including OpenAI, Google DeepMind, and Anthropic, warn of the existential risks posed by artificial intelligence, likening its potential dangers to pandemics and nuclear wars. In a rare consensus, over 350 experts call for urgent regulatory measures and global cooperation to mitigate A.I. threats, underscoring the technology's rapid advancement and its unforeseen societal impacts.

CEOs Alarmed: AI's Existential Threat Within a Decade A striking 42% of CEOs surveyed at the Yale CEO Summit believe artificial intelligence could pose a catastrophic threat to humanity in the next 5-10 years. The survey, reflecting views from diverse industries, reveals a split among business leaders on AI's potential dangers versus its opportunities, highlighting the urgent need for a deeper understanding and strategic approach to AI's rapid advancements.

AI's Dystopian Potential: Five Alarming Predictions from Top Researchers Experts warn of artificial intelligence's capacity to cause catastrophic harm, drawing parallels to species extinction and global disruption. These scenarios range from AI surpassing human

intelligence and manipulating resources to present-day biases in algorithms causing societal injustices, illustrating both immediate and future threats posed by unregulated AI development.

AI's Existential Threat: A call to action from Leading Scientists
Sam Altman, CEO of OpenAI, and other top industry figures issue a stark warning about AI's potential to cause human extinction. This unified statement, emphasizing the urgent need for global prioritization akin to addressing pandemics and nuclear war, reflects a growing consensus among scientists and tech leaders about the imminent risks of unregulated AI development.

AI's Potential Peril: Godfather of AI has Grave Concerns Over Future of Humanity
Geoffrey Hinton, renowned as the 'godfather of AI', expresses his alarm about artificial intelligence surpassing human intelligence, potentially leading to 'the end of people.' While his warnings contrast with the views of other experts who emphasize AI's immediate challenges and benefits, the debate highlights the crucial need for responsible development and regulation of AI technologies.

Treatment: Imminent/Actual Risks

AI Revolution: A Tidal Wave of Job Disruption Looms on the Horizon
The impending wave of artificial intelligence is poised to drastically reshape the global job market, with predictions of significant job displacement and transformation. Amidst the potential for both economic upheaval and unprecedented productivity gains, the need for proactive government and corporate strategies to manage this transition becomes increasingly vital.

Generative AI Fuels Disinformation Surge, Amplifying Global Digital Oppression A new report from Freedom House highlights the alarming use of generative AI by governments worldwide to spread disinformation and intensify internet censorship. This evolving digital strategy, encompassing AI-generated texts, images, and videos, poses a growing threat to internet freedom and public discourse, marking a troubling trend in the manipulation of online information.

AI's Discrimination Dilemma: Severe Risks in Banking Sector Highlighted The increasing use of AI in banking and financial services is raising serious concerns about the amplification of existing human biases, potentially leading to discriminatory outcomes. Experts emphasize the need for comprehensive datasets and unbiased development teams to counter this trend, underscoring the urgency for effective regulatory measures and ethical AI practices in the financial industry.

AI Revolution Could Deepen Global Economic Inequality, Research Warns A study reveals that the rise of artificial intelligence and robotics could exacerbate the economic divide between developed and developing nations, threatening to channel more investment towards advanced economies and potentially replace the labor force in less developed countries. This shift urges policymakers in developing nations to focus on enhancing productivity and skills to avoid being left behind in the global AI-driven transformation.

AI and Deepfakes: A Growing Threat in Cybersecurity Landscape The proliferation of AI and deepfake technology is raising alarms in the IT sector, with concerns about its misuse in fraudulent activities and its impact on businesses. While AI holds the potential for positive contributions, its increasing sophistication in creating synthetic media poses significant risks, calling for heightened awareness and training in cybersecurity measures to combat these emerging challenges.

Treatment: Positive Possibilities

DeepMind's AI Unveils Millions of New Material Possibilities, Revolutionizing Research
DeepMind's latest breakthrough in AI, with the ability to predict the properties of over 2.2 million new materials, marks a significant leap forward in materials science. This innovation could pave the way for advancements in diverse fields such as electronics, batteries, and solar cells, potentially transforming the landscape of modern technology and manufacturing.

AI Offers Breakthrough in Treating Rare Metabolic Disease in Children Leveraging the power of big data, advanced algorithms, and high-performance computing, researchers have utilized artificial intelligence to discover a promising new drug for cystinosis, a rare metabolic disorder. This groundbreaking research, funded by the Swiss National Science Foundation and led by the University of Zurich, brings new hope for effective treatment options for affected children worldwide.

Innovative AI Model Revolutionizes Drug Synthesis, Boosting Efficiency and Sustainability
Researchers from the Ludwig Maximilian University of Munich, ETH Zurich, and Roche Pharma have developed a groundbreaking AI model capable of predicting the optimal chemical alterations in drug molecules. This advancement promises to reduce laboratory experiments significantly, enhancing the efficiency and sustainability of chemical synthesis in pharmaceutical development. The model accelerates the creation of more effective drug variants, marking a significant leap in medicinal chemistry.

AI's Role in Tackling Climate Change: Harnessing Data for Effective Solutions Experts at the Johns Hopkins Institute for Assured Autonomy highlight the significant potential of AI in addressing the complexities of climate change. By analyzing vast climate data sets, AI aids in making informed predictions and deploying timely mitigation strategies. The technology's application ranges from understanding oceanic changes to monitoring environmental shifts via satellites, offering a transformative approach to combating one of the most pressing global challenges.

Study Reveals AI's Positive Impact in Workplace Productivity and Employee Satisfaction
Recent research by a team of economists, including Stanford's Erik Brynjolfsson, highlights the beneficial impact of AI in the workplace, challenging the notion of AI as a job eliminator. Their study, focusing on AI in customer service, demonstrates increased worker productivity, enhanced customer experiences, and reduced employee turnover, showcasing AI as a valuable tool in augmenting human capabilities and improving overall workplace dynamics.

Post-treatment Items

MANIP CHECK.
You were just presented with some news headlines. Please tell us to what extent you agree of disagree with the following statement:

- The headlines I just saw were mostly about existential risks AI may bring
- The headlines I just saw were mostly about the positive potential that AI holds
- The headlines I just saw were mostly about the imminent risks AI poses today

Participants saw the statement corresponding to the treatment group to which they were assigned.

ATTITUDES - LIKERT 1 - 10

Next, you will see a list of various outcomes associated with Artificial Intelligence (AI). For each item listed, we would like you to answer the question below. Remember, there are no right or wrong answers. Your perspectives are invaluable for understanding how the public perceives the potential impacts of AI.

1. How likely do you think it is that AI will cause or be capable of causing the described outcome?

OR

Next, you will see a list of various outcomes associated with Artificial Intelligence (AI). For each item listed, we would like you to answer the question below. Remember, there are no right or wrong answers. Your perspectives are invaluable for understanding how the public perceives the potential impacts of AI.

1. How likely do you believe that the following outcomes related to Artificial Intelligence will occur within the next ten years?

OR

Next, you will see a list of various outcomes associated with Artificial Intelligence (AI). For each item listed, we would like you to answer the question below. Remember, there are no right or wrong answers. Your perspectives are invaluable for understanding how the public perceives the potential impacts of AI.

2. How significant would be the impact of each outcome, if it were to occur?

The order of AI-related outcomes are randomized

1. Existential Risks
   - AI leading to a global catastrophic event.
   - AI making humans obsolete.
   - AI autonomously starting a war.
   - AI causing a significant environmental disaster.

2. Immediate/Actual Risks
   - AI leading to significant job losses in certain sectors.
   - AI being used in mass surveillance systems.
   - AI increasing the spread of misinformation online.
   - AI making decision by organizations (public and private) more biased .

3. Positive Possibilities
   - AI improving healthcare through better diagnosis and treatment.
   - AI enhancing education through personalized learning.
   - AI reducing human error in critical tasks.
   - AI contributing to solving complex environmental issues.

Rating Scale:

Likelihood 1

1. Very Unlikely
2. Unlikely
3. Neutral
4. Likely
5. Very Likely

Likelihood 2

1. Extremely Unlikely
2. Very Unlikely
3. Moderately Unlikely
4. Slightly Unlikely
5. Neutral
6. Slightly Likely
7. Moderately Likely
8. Likely
9. Very Likely
10. Extremely Likely

Impact

1. Extremely Insignificant
2. Very Insignificant
3. Moderately Insignificant
4. Slightly Insignificant

5. Neutral
6. Slightly Significant
7. Moderately Significant
8. Significant
9. Very Significant
10. Extremely Significant

DEBRIEFING.
Thank you for your participation in this research study. Now that you completed or have ended your participation, we will provide you with some additional information about the purposes of this study.
What you should know about this study
The main purpose of this study was for us to observe how participants feel about new technological developments, such as AI and ChatGPT. Participants in this study have been exposed to various headlines about AI, some highlighting the more positive aspects of AI development, some highlighting more negative aspects of it. It is important to realize that the headlines you have been exposed to, may not be an accurate representation of the variety in which the media covers AI related issues.

If you have questions
The main researchers conducting this study are [names and contact details of researchers]. If you have questions, you may contact one of the main researchers. If you have any questions or concerns regarding your rights as a research participant in this study, you may contact the Ethics Committee.

[contact details Ethics Committee]

Existential Risk Narratives About Artificial Intelligence Do Not Distract From Its Immediate Harms: Populated Pre-Analysis Plan

This document serves as a Populated Pre-Analysis Plan to complement our pre-registrations. A populated PAP is a short, publicly available document that includes all completed analyses and explains any deviations or infeasibilities (Banjaree et al. 2020). This ensures transparency while allowing the research paper to focus on presenting meaningful findings. The relevant pre-registrations and analyses can be found under the following links:

- PAP Study 1: https://bit.ly/PAP-S1
- PAP Study 2: https://bit.ly/PAP-S2
- PAP Study 3: https://bit.ly/PAP-S3
- Results of all pre-registered analyses: https://bit.ly/AI_narratives_full_results

We provide a detailed account of the findings from our three studies, highlighting how they informed adjustments to our measures, variables, and analyses. While the relevant pre-registrations for each study document the original hypotheses and changes between the three studies, this document focuses on how these changes were driven by our evolving understanding of the distraction hypothesis, the core focus of our research. The distraction hypothesis posits that existential risk narratives about AI may shift attention away from AI's more immediate risks. Specifically, it suggests that exposure to information emphasizing existential risks reduces individuals' perceptions of the capability, likelihood, and impact of more immediate, tangible risks associated with AI. By complementing the pre-registrations, this Populated PAP ensures full transparency and reflects our commitment to open science practices.

We pre-registered a large number of hypotheses and variations in outcome measures for this study. This reflects an ambitious but ultimately unwieldy attempt to capture a broad spectrum of potential effects. This Populated PAP serves as an account of how our findings informed the adjustments made across studies, ensuring transparency in documenting the evolution of our research in response to emerging insights.

For Study 2 and Study 3, which built on Study 1, we created new pre-registrations to document updated hypotheses and plans, reflecting the iterative nature of our research. These pre-registrations captured adjustments informed by the findings of previous studies while maintaining transparency about the evolution of our expectations and methods. For instance, following the results of Study 1, we refined the distraction hypothesis by adjusting its direction in Study 2 to reflect emerging evidence. In Study 3, we reintroduced the capability measure from Study 1 and combined it with the key measures from Study 2, thus making a distinction between "capability," "likelihood," and "impact" of AI risk. We did so because Study 1 and Study 2 led us to recognize the original "capability" variable in Study 1 as potentially capturing different constructs. These adjustments were carefully documented and justified within the updated pre-registrations for each subsequent study to ensure accountability and transparency.

In our manuscript, we focused on the variables most relevant to the distraction hypothesis to focus on coherent results that remain grounded in our original research goals. The

distraction hypothesis is central to our research, as evidenced by Study 1, and the subsequent studies were designed to build on this foundation. By concentrating on the overlapping variables “capability”, “likelihood”, and “impact”, we chose to prioritize the outcomes that allow for meaningful comparisons and insights across studies. This approach ensures a coherent presentation of the findings while maintaining fidelity to our original intentions as outlined in the pre-registrations.

This Populated PAP complements our pre-registrations by serving as a comprehensive account of how our findings informed adjustments to the measures, variables, and analyses across studies. While we applied a necessary focus in the manuscript, prioritizing the main results around the distraction hypothesis, we maintained our original frame and intentions. We align with Banerjee et al. (2020) by using our pre-registration in combination with this Populated PAP to ensure transparency about deviations and the rationale behind them.

Thought Processes Behind Additions and Omissions

Across the three studies, we pre-registered a total of 69 hypotheses: 18 in Study 1 (6 main, 12 additional), 27 in Study 2 (18 main, 9 related to mechanisms), and 24 in Study 3. While this approach allowed us to explore multiple ways of assessing key constructs, it also led to an ambitious but ultimately unwieldy design that required refinement in subsequent studies.

Study 1
In Study 1, we pre-registered 18 hypotheses, including 6 main hypotheses (of which Hypothesis 1a was the distraction hypothesis) and 12 additional ones. These hypotheses focused on outcomes such as perceived capability of AI risks and perceptions of AI’s immediate versus existential harms. While this broad approach provided a strong foundation for examining the distraction hypothesis, one key limitation became apparent during analysis: the “capability” measure appeared to conflate two distinct constructs—likelihood and impact of AI risks. This conflation limited the interpretability of the results and highlighted the need to refine and separate these measures in subsequent studies. Additionally, we were concerned that our use of a 1–5 scale for key variables might have resulted in ceiling effects, which could have reduced the sensitivity of our measures. These findings informed the adjustments made in Study 2.

Study 2
For Study 2, we pre-registered 27 hypotheses, comprising 18 main hypotheses and 9 focusing on mechanisms related to the distraction hypothesis. A new pre-registration was created to document several important adjustments:

- **Dissecting Measures:** To address the conflation of likelihood and impact of AI identified in Study 1, we split the “capability” measure into two distinct variables: likelihood of AI risks and impact of AI risks. This allowed us to analyze these constructs separately, providing clearer insights into participants’ perceptions of AI risks.
- **Addressing Potential Ceiling Effects:** To capture a broader range of responses, we changed the scale for key variables from a 1–5 scale (used in Study 1) to a 1–10 scale. This adjustment reduced any ceiling effects and improved the sensitivity of our measures.

- **Exploring Mechanisms:** We introduced 9 new hypotheses to examine potential mechanisms underlying the distraction hypothesis. These mechanisms included variables such as AI's perceived power, fears about AI's potential power, and policy preferences for regulating AI. These additions aimed to deepen our understanding of the processes driving the observed effects.
- **Omitting Conspiracy Theories:** The belief in conspiracy theories, a variable from Study 1, was excluded in Study 2 due to its lack of significant effects. This decision allowed us to focus on refining and expanding measures that were more directly relevant to AI risk perceptions.

The pre-registration for Study 2 transparently captured these refinements and additions, ensuring that all changes were clearly documented.

Study 3

In Study 3, we pre-registered 24 hypotheses. This new pre-registration aimed to consolidate and integrate the measures from both prior studies into a single, comprehensive design, ultimately shaping the focus of our manuscript. Key adjustments included:

- **Reintegrating and Expanding Measures:** Study 3 included all three outcome variables—capability, likelihood, and impact—to capture the full scope of participants' perceptions of AI risks. By incorporating these distinct constructs together, we ensured a comprehensive evaluation of the distraction hypothesis.
- **Including Both Scales:** To combine insights from Studies 1 and 2, we included both the 1–5 scale (used in Study 1) and the 1–10 scale (introduced in Study 2). This allowed us to maintain completeness and comparability across studies while addressing the limitations identified earlier.
- **Omitting Mechanisms:** The mechanism variables introduced in Study 2 (e.g., AI's perceived power and policy preferences) were omitted in Study 3 because we found no significant effects in Study 2. Instead, we chose to focus on refining the core outcomes related to the distraction hypothesis to ensure a more streamlined and targeted analysis.
- **Retaining Core Variables:** Consistent with our focus on the distraction hypothesis, we retained all key variables from Studies 1 and 2 that directly assessed the hypothesis, ensuring continuity and comparability.

All of these adjustments are documented at the top of the pre-registrations for each study.

References

Banerjee, Abhijit, Esther Duflo, Amy Finkelstein, Lawrence F. Katz, Benjamin A. Olken, and Anja Sautmann. 2020. "In Praise of Moderation: Suggestions for the Scope and Use of Pre-Analysis Plans for RCTs in Economics." NBER Working Paper N. 26993. doi:10.3386/w26993.
